# The Human Touch: Skin Temperature during the Rubber Hand Illusion in Manual and Automated Stroking Procedures

**DOI:** 10.1371/journal.pone.0080688

**Published:** 2013-11-18

**Authors:** Marieke Rohde, Andrew Wold, Hans-Otto Karnath, Marc O. Ernst

**Affiliations:** 1 Department of Cognitive Neuroscience, University of Bielefeld, Bielefeld, Germany; 2 Cognitive Interaction Technology Centre of Excellence, University of Bielefeld, Bielefeld, Germany; 3 Multisensory Perception & Action Group, Max Planck Institute for Biological Cybernetics, Tübingen, Germany; 4 Division of Neuropsychology, Hertie-Institute for Clinical Brain Research, Tübingen, Germany; 5 Research Division Mind and Brain, Charité - Universitätsmedizin Berlin, Berlin, Germany; ICREA-University of Barcelona, Spain

## Abstract

A difference in skin temperature between the hands has been identified as a physiological correlate of the rubber hand illusion (RHI). The RHI is an illusion of body ownership, where participants perceive body ownership over a rubber hand if they see it being stroked in synchrony with their own occluded hand. The current study set out to replicate this result, i.e., psychologically induced cooling of the stimulated hand using an automated stroking paradigm, where stimulation was delivered by a robot arm (PHANToM^TM^ force-feedback device). After we found no evidence for hand cooling in two experiments using this automated procedure, we reverted to a manual stroking paradigm, which is closer to the one employed in the study that first produced this effect. With this procedure, we observed a relative cooling of the stimulated hand in both the experimental and the control condition. The subjective experience of ownership, as rated by the participants, by contrast, was strictly linked to synchronous stroking in all three experiments. This implies that hand-cooling is not a strict correlate of the subjective feeling of hand ownership in the RHI. Factors associated with the differences between the two designs (differences in pressure of tactile stimulation, presence of another person) that were thus far considered irrelevant to the RHI appear to play a role in bringing about this temperature effect.

## Introduction

When an observer receives synchronous stroking on a hand that is occluded from vision and a rubber hand that is placed where their hand would naturally lie (on the same side), they often perceive that the stroking they feel originates from the rubber hand [1]. This Rubber Hand Illusion (RHI) leads observers to believe that the rubber hand could be part of their own body. 

Vividness ratings (e.g., [2]) or questionnaires (e.g., [1],[ 3]) can be used to put this compelling subjective feeling of ownership over the rubber hand in numbers. Sometimes, however, one would prefer to use objective or implicit measures of this illusion in order to exclude cognitive biases, and such objective measures are not easy to find. Among the behavioural and physiological variables that have been reported to correlate with the RHI are: A drift of proprioceptively sensed body position towards the rubber hand (proprioceptive drift; e.g., [1,4]), skin conductance response to threat (e.g., [5]), visuotactile cross-modal congruency effects (e.g., [6]), processing time of tactile stimuli [2], time of onset of the illusion (e.g., [7]), rate of self recognition [8], local histamine reactivity [9], and neural activity in a number of brain areas (e.g., [7,10,11]). Yet, for many of these correlates, confounding variables have been identified. For instance, proprioceptive drift appears to be due to visuo-proprioceptive conflict rather than ownership over the rubber hand (e.g., [12,13]), and rubber hand ownership as well as drift can occur without tactile stimulation at all [14]. Similarly, slowing down of tactile processing was also observed under visuo-proprioceptive conflict without the subjective RHI [15]. An increased skin conductance response to threat can also be measured when stroking a table top (even if this effect still correlates with perceived body ownership over the table top [5]). Such findings suggest that the variables in question are not strict correlates of the illusion of ownership over a rubber hand but instead relate to factors in the procedure that are at best necessary but not sufficient to induce the RHI (cf. [13] for a discussion of ways to quantify the RHI).

In 2008, Moseley et al. [2] reported a new physiological correlate for the illusion of ownership in the RHI. In a series of experiments, they showed that in the RHI, the stimulated hand was cooler than the non-stimulated hand, and that this difference in temperature was correlated with the reported intensity of the subjective feeling of ownership over the rubber hand [2]. This result falls in line with a large body of research that establishes links between skin temperature, body ownership, tactile processing and pain perception, as discussed in 2. Furthermore, Kammers et al. [16] showed that the amount of proprioceptive drift in the RHI can in turn be modulated by manipulating the hand temperature of a participant in the RHI, even though this modulation did not involve significant changes in subjective experience of ownership. As a side result, this study replicated Moseley et al.’s [2] results that synchronous stroking leads to a relative cooling of the stimulated hand. Skin cooling after congruent visuo-tactile stimulation has also been reported for a full body variant of the experiment where tactile vibrators were used to deliver tactile stimulation (out of body experience, [17]). There are, however, also studies that call a direct link between subjective ownership and skin cooling into question. Hohwy and Paton [18] replicated the cooling effect in a variant of the basic RHI, but did not find corresponding temperature drops in further variations of the paradigm that involved equal levels of subjective disownership of the participants’ hand. In a study with with two participants with hand or arm amputations, Marasco et al. [19] reported conflicting results on body temperature (both cooling and warming under visuo-tactile synchrony in one participant; no effect in the other). The absence of a clear result in this latter study, which focused on much clearer subjective and perceptual measures, may however simply be due to a small sample size. 

Taking all these results together, there are still reasons to believe that, compared to other measures, the “psychologically induced cooling” [2] of the hand may be a more strict correlate of the subjective feeling of ownership, as a direct causal link is proposed: The feeling of ownership over the rubber hand leads to disownership of one’s own hand, which in turn causes the relative cooling [2]. 

The aim of the research presented here is to investigate the skin cooling during the RHI more closely. In Experiment 1 and 2, we set out to replicate two of Moseley et al.’s [2] results in an experimental setup, where two robot arms provide the tactile stimulation [12]. We focused on changes in temperature over time. Given that these attempts to replicate the cooling effect reported by Moseley et al. [2] were unsuccessful, we then reverted to a manual stimulation setup in Experiment 3. There we could observe the characteristic skin cooling reported in [2] in both the experimental and in the control condition. The subjective feeling of ownership over the rubber hand, by contrast, was linked to visuo-tactile synchrony in stimulation in all three experiments. We thus conclude that hand cooling is not a strict correlate of the feeling of ownership in the RHI and point out possible confounding variables associated with this cooling effect and the procedures used here. 

Participants in all three experiments were naïve to the purpose of the study and received a small fee of 8 €/h for their participation. All participants provided written consent before participating in this study. The study was approved by the ethics committee of the department of medicine at the University of Tübingen and was conducted in accordance with the Declaration of Helsinki. The results from all three experiments are published as a .mat (Matlab) file alongside a text document explaining the format in the compressed (.zip) folder File S1.

## Experiment 1: Time Course of Hand Cooling (Automated Stroking)

The objective of Experiment 1 was to replicate Moseley et al.’s [2] Experiment 1, where a cooling of the experimental hand (relative to control) was observed. Additionally, we measured the time course of hand temperature continually, to investigate the time course of changes in hand temperature.

### Materials and Methods

#### Participants

18 healthy participants took part in this study (age range: 19-28; median age: 24; 11 females; all participants right-handed, as reported with the Edinburgh Handedness Inventory [20], 10 item version). 

#### Experimental Setup

**Figure 1:**
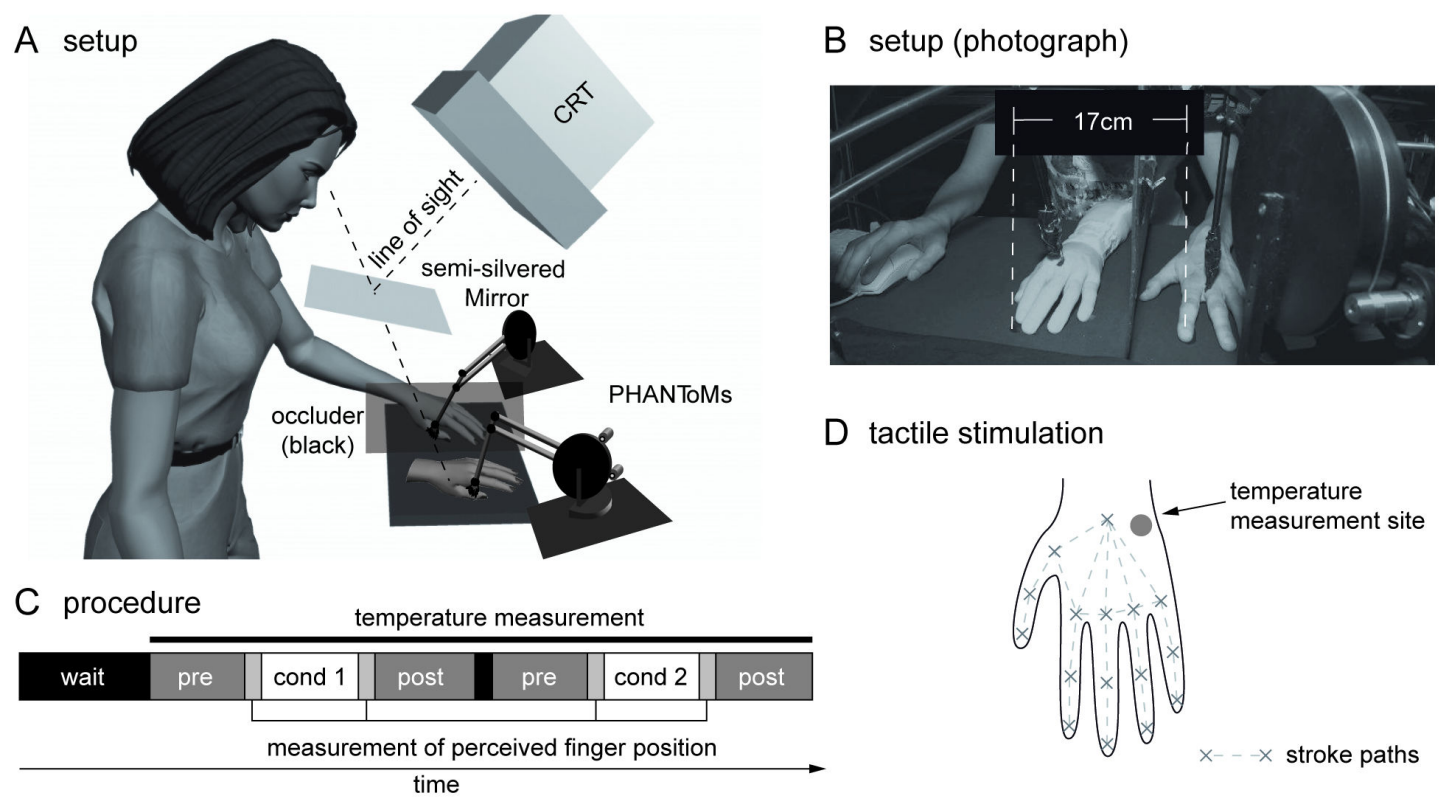
The RHI setup used in the study. Diagram (A) and photograph (B) of the setup. Two PHANToM^TM^ force-feedback devices with paintbrush endings stroke the participant's occluded hand and the visible rubber hand. For testing proprioceptive drift, a probe dot is projected into the visual field using the CRT monitor and a semi-silvered mirror. The participant in (B) has given written informed consent, as outlined in the PLOS consent form, to publication of their photograph. C: The experimental procedure. D: Calibration points and lines along which strokes were applied, as well as temperature measurement location.

The experiment was conducted using the same computer controlled setup used in our previous work [13]. Two PHANToM^TM^ force-feedback devices (SensAble Technologies Inc) served as robot arms to stroke both the participant's and the rubber hand with custom-made paintbrush endings (Figure 1). A realistic left rubber hand was placed 17 cm to the right of the participant’s real left occluded hand, such that the middle finger of the rubber hand was aligned with the body midline. Participants could see the rubber hand only for the duration of the stimulation. Otherwise the room was dark. 16 points of the hand (Figure 1D) were matched between the rubber hand and the participants’ hand and strokes were applied between neighbouring points (500ms stroking, 500ms pause in between). In the synchronous condition the strokes on the rubber hand and real hand were both spatially and temporally aligned. As a control condition, we chose asynchronous stroking, where strokes were spatially random between the hands and temporally out of phase.

Hand temperature was recorded continually at 1 Hz with an infrared thermometer (Voltcraft IR 1020-50D) mounted to the setup throughout the experiment. Recordings were made on a fixed spot by the wrist (cf. Figure 1D). In order to record perceived hand location, a white probe dot was projected into the visual field using the CRT and a semi-silvered mirror while the room was dark (see 13 for more details of the setup and measurement of perceived finger location). Participants could adjust the position of the dot to the felt horizontal location of their left index finger using the scroll wheel of a mouse with their right hand.

#### Procedure

Figure 1C depicts the timeline of the experiment. Participants were seated for approximately 10-15 min prior to testing in order to adjust to room temperature. During this time they read and signed the consent forms. The measurement of hand temperature started 200 s before the experimental procedure was initiated and continued throughout the experiment. During the experiment, the room lights were turned off. Participants first had to indicate their perceived location of the left index finger in darkness by adjusting the horizontal position of a projected dot (cf. “Setup”) for 3 trials. Then participants received 200 s of either synchronous or asynchronous stroking, during which the LED in the setup was switched on, so participants could see the rubber hand being stroked. After this, they again had to adjust the position of the dot for 3 trials. This was followed by 200 s post experiment temperature recording with the lights on. After such a block, the participants were asked to evaluate the vividness of their experience of ownership over the rubber hand using an 11 point Likert scale (0-10; 0 being strongly disagree, 10 being strongly agree), with the following question; “I felt as though the rubber hand could have belonged to my own body”, which we had found to be a good indicator of vividness during piloting (cf. also [3], very similar questionnaire item 5). The procedure of using a single questionnaire item was copied from [2], as only a rough estimate of the occurrence of the subjective feeling of ownership was required. The vividness measure was asked in English or German, according to language proficiency. Participants were exposed to both conditions (synch/asynch) within one session, with a short break between the blocks (cf. Figure 1C). The order of stroking condition was counter-balanced across participants.

#### Analysis

All results were analyzed using Matlab R2010 (functions signrank, ttest, and corr from the Statistics Toolbox; function rm_anova2 by Schurger from the Matlab Central File Exchange [21]). 

### Results

**Figure 2:**
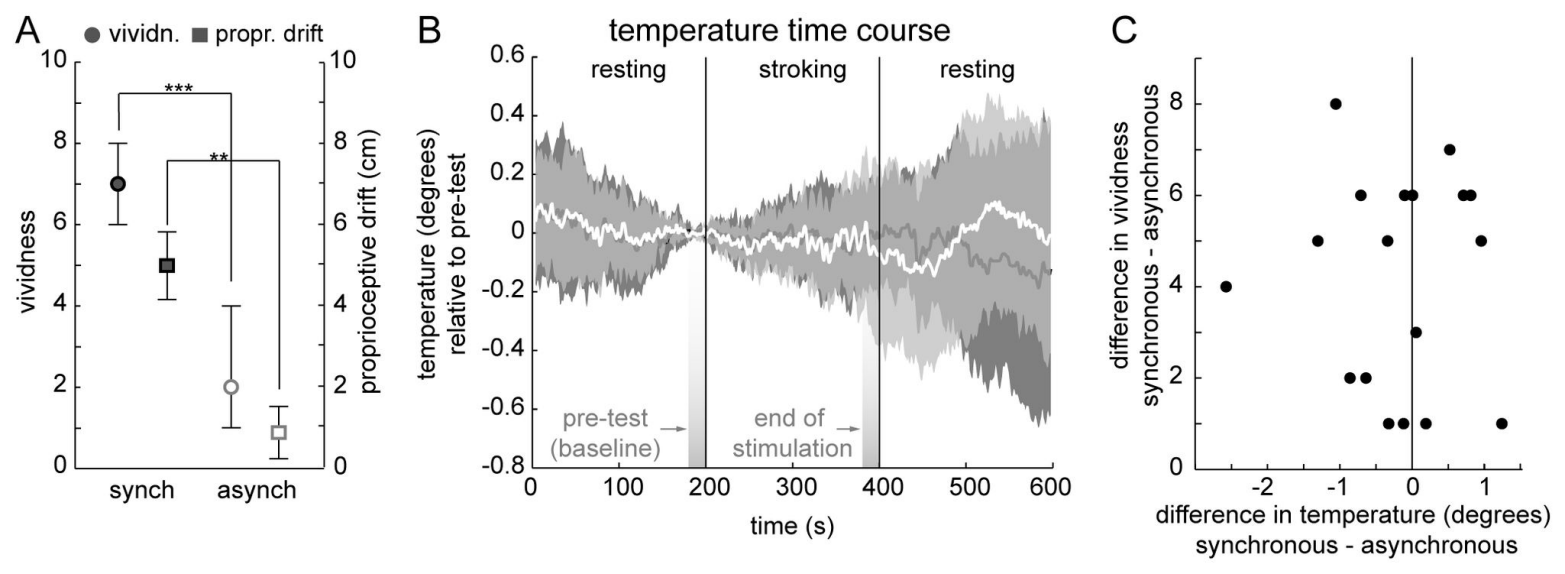
Results Experiment 1. A: Proprioceptive drift (left y-axis; mean and SEM) and vividness ratings (right y-axis; median and 25 and 75 percentile). B: Temperature in the synchronous (dark) and asynchronous (white) conditions across time. Temperature is given relative to temperature at the beginning of the stroking (pre-test at 200 s) to accentuate possible trends due to the onset of stroking, which is why the time lines converge at this point. For clearer visualization in this Figure, the temperature timelines were smoothed using a Gaussian filter (window size: 7). Median and symmetrical IQR. C: Differences in vividness against differences in temperature at the end of stimulation for individual subjects.

We observed the differences in vividness and proprioceptive drift usually reported in the literature when comparing synchronous and asynchronous stroking in the RHI (Figure 2A; Wilcoxon signed rank test vividness: p<0.001, rank=0; paired-sample t-test proprioceptive drift: t(17)=3.50, p=0.003). However, there were no significant effects on hand temperature between conditions or across time (cf. Figure 2B). Hand temperature was compared before the onset of tactile stimulation and at the end of tactile stimulation (average over 20 s, see Figure 2B) using a two-way repeated measures ANOVA with factors test phase (pre-test vs. end of stimulation; average over 20 s, cf. Figure 2B) and condition (synch vs. asynch). There were no significant main effects for either test phase (F(1,17)=0.65, p=0.431) or condition (F(1,17)=0.76, p=0.396) and no interaction (F(1,17)=0.11, p=0.746; full ANOVA table in Table S1 in File S1). Also, no correlation between differences in vividness of the RHI and differences in temperature were observed (Figure 2C; correlation: Spearman’s ρ=-0.02, p=0.951).

These results replicate previous findings on vividness and proprioceptive drift in the RHI, but did not show any discernable pattern for any direction of temperature change or how it relates to vividness of the subjective feeling of ownership. However, in most of their experiments, Moseley et al. [2] quantified the cooling as a relative cooling between hands and conditions. If the effect is comparably subtle, a comparison between hands can help to correct for some of the noise in the change of hand temperature. 

## Experiment 2: Temperature Differences between Hands (Automated Stroking)

The objective of Experiment 2 was to replicate Moseley et al.’s [2] Experiment 3, where the authors had used a longer time of stimulation (7 min) than we did in our Experiment 1. The authors had also used a comparison of temperature between both hands at the end of stroking, rather than to look for temperature changes over time within the stimulated hand. This between-hand approach could help filter out general body heating or cooling trends that may have added noise to the effect in our Experiment 1. 

### Materials and Methods

#### Participants

16 new healthy participants took part in the experiment (age range: 18-31; median age: 24; 8 females; all subjects were right-handed). 

#### Procedure

The procedure during this experiment was identical to the previous except for the total stimulation duration (now a total of 7 min for stroking) and a modification of the temperature measurement protocol. Instead of measuring temperature continually, temperature recordings were taken manually from identical spots on both hands after 5 min of tactile stimulation (6 times during the last 2 min of stroking, i.e., every 20 s). Each of these measurements from one hand comprised of 6 consecutive recordings. This was repeated, alternating between the hands until the tactile exposure was completed. The 3x6 measurements for each hand and condition were then averaged, i.e., there was one resulting value per hand and condition. Finger proprioception was recorded before and after stroking as in Experiment 1. Block order and the order of measured hands were counter-balanced.

### Results

**Figure 3:**
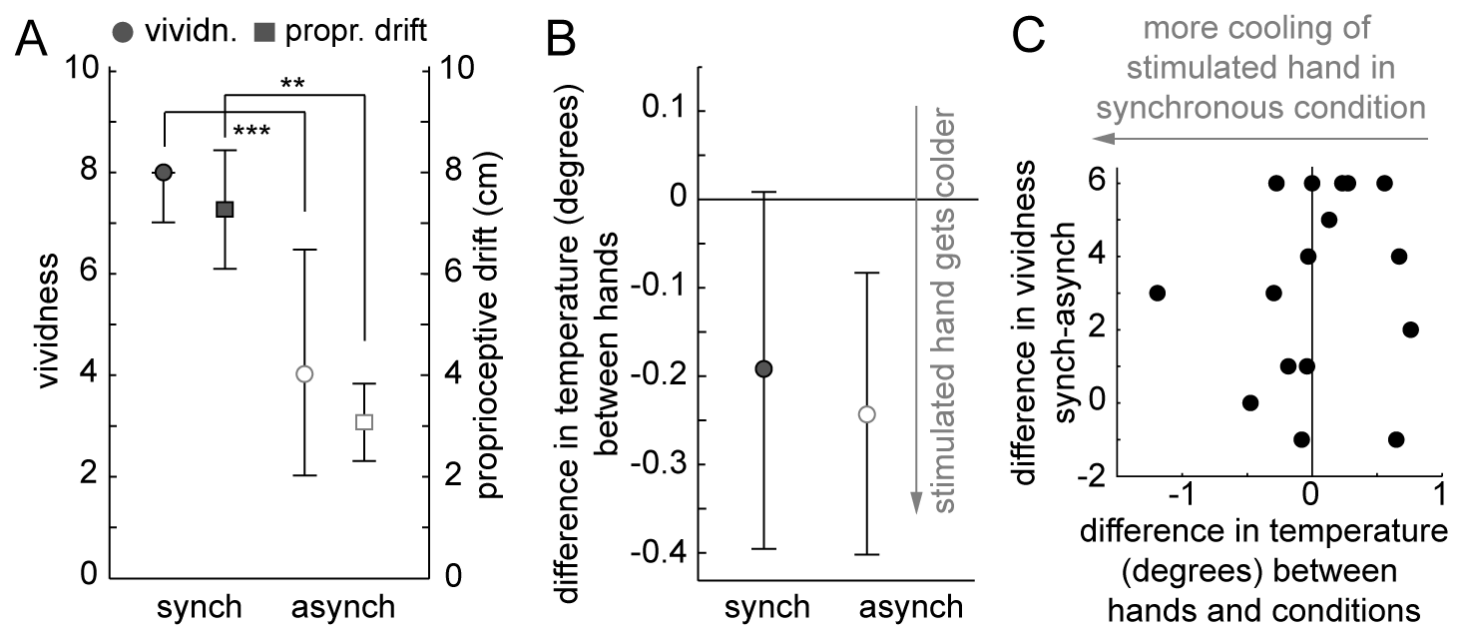
Results Experiment 2. A: Proprioceptive drift (left y-axis; mean and SEM) and vividness ratings (right y-axis; median and 25 and 75 percentile) and. B: Differences in temperature between hands (mean and SEM). C: In individual participants, differences in temperature between hands and conditions (x-axis) is not correlated with differences in vividness between conditions (y-axis); Spearman’s ρ=0.22, p=0.406.

We were able to replicate the vividness and drift results of the previous study (Figure 3A; Wilcoxon signed rank test vividness: p<0.001, rank=5; paired-sample t-test proprioceptive drift t(15)=3.35 p=0.004). The temperature (Figure 3B) was analyzed using a two-way repeated measures ANOVA with factors: condition (synch/asynch) and hand (stimulated/non-stimulated). There was no significant interaction between hand and condition (F(1,15)=0.16 p=0.698) and no main effect for either condition (F(1,15)=0.01 p=0.942) or hand (F(1,15)=1.51 p=0.239; full ANOVA table in Table S1 in File S1) and no correlation between changes in temperature and changes in vividness (Figure 3C; statistical test results in caption).

We had expected an interaction between the factors condition and hand as in Moseley et al. [2], but observed no significant differences in hand temperature at all. One important difference between our study and the mentioned study was that we used automated stroking with a paintbrush, rather than manual [2,18] or paintbrush [16] stroking by another person.

## Experiment 3: Temperature Differences between Hands (Manual Stroking)

The objective of Experiment 3 was to replicate Moseley et al.’s [2] Experiment 2, where the control condition did not involve any visual or tactile stimulation and which had the most significant result. For this experiment, we tried to copy the manual stroking procedure described in [2] as faithfully as possible, including the experimental setup, to test whether the failure to replicate the result is due to our automated stroking setup.

### Materials and Methods

#### Participants

18 new healthy participants took part in this study (age range: 19-28; median age: 22; 12 females; all participants were right-handed). One 19^th^ participant had to be removed from the data set because of an experimental error within the first block. 

#### Experimental Setup

**Figure 4:**
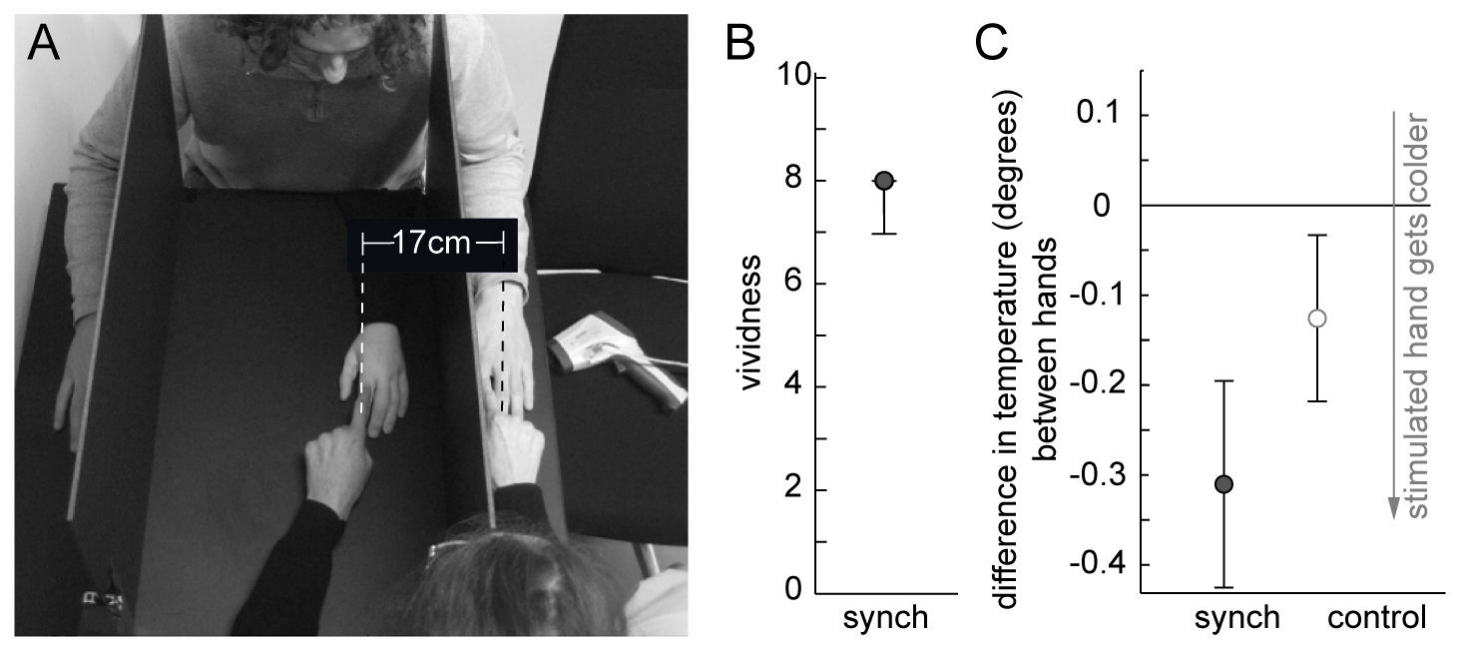
Set-Up and results Experiment 3. A: Picture of the experimental setup. The participant depicted has given written informed consent, as outlined in the PLOS consent form, to publication of their photograph. B Vividness ratings (median and 25 and 75 percentile) synchronous condition. C: Differences in temperature between hands (mean and SEM).

Two partitions were placed on a table so that subjects were unable to view their hands. The left index finger of the participant was positioned onto a marker that was aligned with the index finger of the rubber hand at a distance of 17 cm (Figure 4A). Participants were to sit as close to the table as possible to provide the best view over the rubber hand. The participants sat across from the experimenter who performed the RHI stroking via finger to hand contact. The rubber hand could be easily removed, leaving a tape marker corresponding to the position of the rubber hand’s index finger. 

#### Procedure

The experimenter applied strokes manually (with the fingertip on the participant’s back of the hand and the back of the rubber hand) at a frequency of approximately 1 Hz during the synchronous stroking condition. The experimenter tried to mimick the randomness of stroke location and approximate duration of strokes (500 ms) of the automated setup (naturally, there was some variation in this). As in Moseley et al.’s [2] Experiment 2, during the control block, the rubber hand was removed, and the participant received stroking only on their own hand and were asked to focus their gaze on the tape marker corresponding to the position of the rubber hand’s index finger. Otherwise, the procedure was identical to Experiment 2, beside the fact that perceived hand location was not recorded before or after stroking (the emphasis of this experiment was on the replication of the temperature drop) and vividness was only reported after the synchronous stroking block, as no rubber hand was visible during the control condition. In 4 participants, the hand temperature was measured with a different thermometer by the same manufacturer (Voltcraft IR 900-30S) due to loss of the original measurement device.

### Results

Mean vividness reported for this RHI setup were consistent with other successfully induced RHI experiments (Figure 4B; median=8, range 3-9). The temperature was analyzed identically to the previous experiment. There was no main effect for condition (F(1,17)=1.3, p=0.272), but there was a main effect for hand (F(1,17)= 5.9, p=0.027), indicating a relative cooling of the stimulated hand independent of condition (the stimulated hand was 0.22±0.07 degree colder; mean and SEM). This cooling was on average stronger in the synchronous stroking condition (0.19±0.11 degree difference, mean and SEM), but this interaction between hand and condition was not significant (F(1,17)= 3.0, p=0.103; see Figure 4C; full ANOVA table in Table S1 in File S1). 

**Figure 5:**
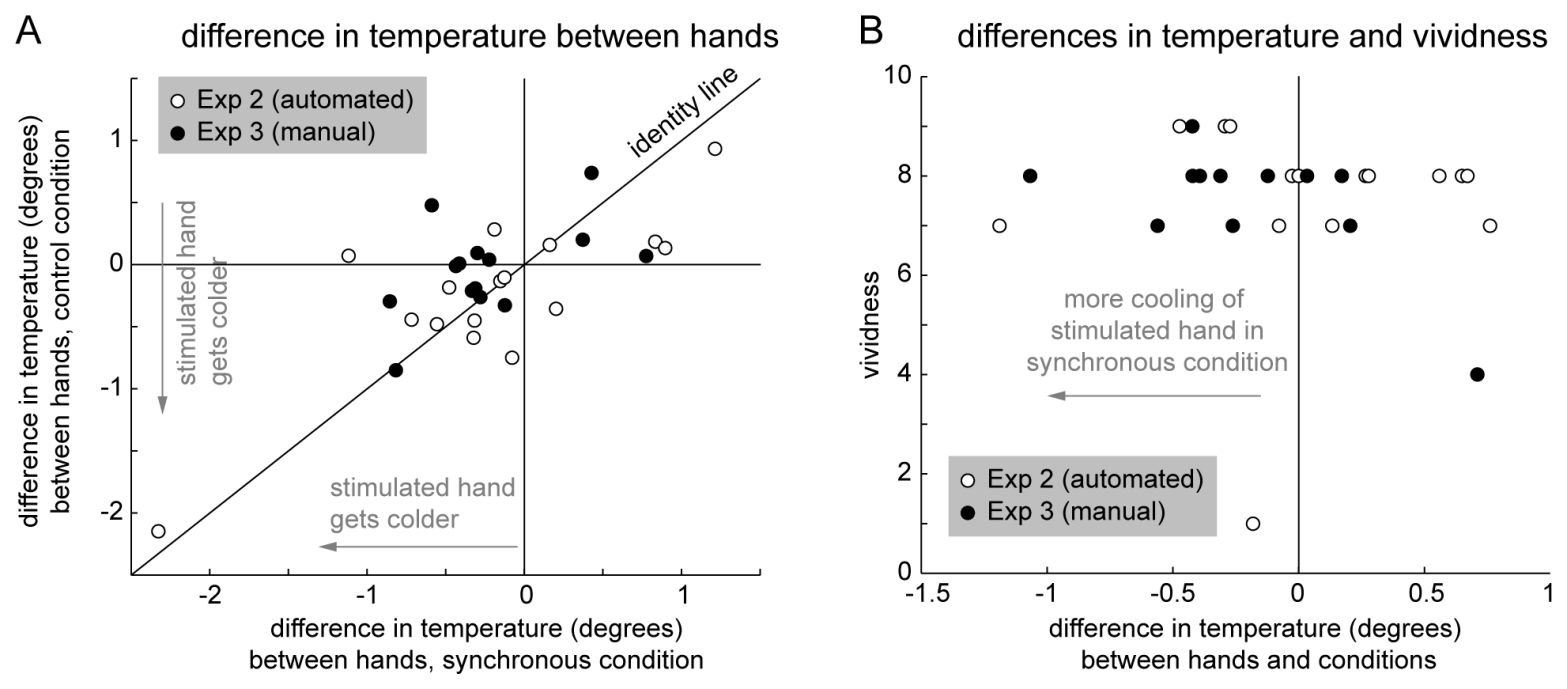
Comparison results Experiment 2 and 3. A: Temperature differences between hands for both conditions and experiments for individual subjects. B: Temperature differences between hands and conditions (x-axis) vs. vividness for individual subjects in the synchronous stroking condition (y-axis) in both experiments. There is no correlation between the two measures in either of the experiments (Exp. 2: Spearman’s ρ=-0.17, p=0.517; Exp. 3: Spearman’s ρ=-0.29, p=0.241; for this plot and analysis, raw vividness ratings were used because there were no vividness ratings for the control condition of Exp. 3; see Figure 3 for an analysis of differences in vividness vs. differences in temperature in Exp. 2).

Figure 5 A depicts the relative cooling between hands in both Experiment 2 and Experiment 3 for all participants and conditions. There is a tendency for a general relative cooling of the stimulated hand in both experiments (more data points in bottom left quadrant).

In Moseley et al.’s [2] experiments, the vividness of participants had also been correlated with the temperature difference between hands. We did not find such correlation effects in either Experiment 2 or Experiment 3 (see Figures 3C and 5B, statistical results in Figure captions).

With the manual stroking setup, we were able to observe a relative cooling of the stimulated hand like in [2] in both the experimental condition and the control condition. 

## Discussion

We set out to replicate the psychologically induced cooling of the stimulated hand during the RHI reported by Moseley et al. [2] in three consecutive experiments, comparing different experimental procedures. While we could not replicate the results at all with the automated stroking procedure (used in [13]), it was possible to replicate the result using a manual stroking procedure, albeit in both the experimental and the control condition. The relative cooling of the stimulated hand in Experiment 3 was of a similar magnitude as the cooling effects reported by Moseley et al. [2], Kammers et al. [16], and Hohwy and Paton [18]. There was also a non-significant trend for this cooling effect to be stronger under synchronous stimulation than in the control condition in Experiment 3. This suggests that the local cooling observed in Experiment 3 may be caused by the same mechanism that also caused relative cooling in previous RHI experiments ([2,16,18]).

Crucially, the differences between experimental procedures did not involve differences in vividness of the subjective RHI, which was always high in the synchronous stroking condition and low in the asynchronous stroking or no hand control condition. There was no correlation between vividness of experienced hand ownership and temperature changes (Figure 5B). In Experiments 1 and 2, also, the usual differences in perceived location of the hand were observed despite an absence of the cooling effect. This shows that this cooling effect requires a specific experimental procedure and is thus not strictly `psychologically induced’ by the subjective RHI and the consequent experienced disownership of one’s real hand, as proposed in [2]. We observe both, a subjective ownership illusion without cooling (Experiment 1 and 2) and a cooling of the stimulated hand independent of subjectively felt ownership (Experiment 3 control). 

The relative cooling only occurs in the manual stroking procedure (Experiment 3) and is independent of the subjective ownership illusion. It is not clear why the replication attempt of Moseley et al.’s [2] result in the automated setup was unsuccessful, or why relative cooling of the stimulated hand occurs in both the experimental and the control condition in our manual setup, despite efforts to exactly replicate Moseley et al.’s [2] procedure. In the following, we will mention a number of possible reasons associated with the different setups, without endorsing any one of them.

- *Social context*. In principle, it is possible that the presence of another person explains the differences between setups, as social or empathic factors have been reported to be involved in the RHI [22]. Neural activation has also been shown to vary depending on whether the source of tactile stimulation is another person (interpersonal touch) or an object (mechanistic touch; cf. [23]).
- *Force*. The paintbrushes used in the automated setup (Experiment 1 and 2) are very soft, and exert a lighter touch than manual stroking.
- *Lighting*. Different lighting conditions were used in the two setups (LED illumination under glass in Experiments 1 and 2; fully illuminated room in Experiment 3).
- *Irregularity*. The touch by an experimenter can be expected to be more irregular in timing and pressure than that of a robot arm executing a program.
- *Unconscious Bias*. Unconscious differences in the amount or manner of stroking between experimental and control conditions may play a role in the case of manual but not automated stroking. For instance, stroking just one hand requires different motor planning by the experimenter than either synchronous or asynchronous bimanual movments and this may lead to differences in the amount, timing and intensity of tactile stimulation delivered.
- *Arousal*. Scenarios may differ in how interesting or exciting stroking in combination with visual inputs is for the participant (this factor is inherently difficult to control for).

Could the cooling effect be a confound due to uncontrolled low level properties of multisensory stimuli like those mentioned above? Illusions like the RHI are not just cognitive or affective illusions but also multisensory illusions, and the dependent variables can be modulated by seemingly irrelevant low level stimulus properties. Manual stroking procedures will always be less well controlled and more prone to such confounds (biases, experimenter effects, social context, etc.). However, Salomon et al. [17] recently reported a body-wide cooling effect in a full-body version of the illusion for automated spatially and temporally congruent visuo-tactile stimulation (compared to temporally congruent, but spatially incongruent stimulation). Their study controlled for most of the mentioned potential confounds, with the exception of arousal. Such well-controlled procedures are essential in explaining the generative mechanisms of body image and its illusions that tend to rely on interaction of processes on different levels.

The results do not offer an explanation for why Moseley et al.’s [2] result was not replicated here using the automated procedure. Yet, they show a clear dissociation of subjectively felt ownership and the cooling effect. Hohwy and Paton [18] reported a similar result, where a cooling effect occurred only in one out of three manipulations that all involve equal levels of subjectively felt disownership of the own hand. It may also be worth mentioning that Moseley et al. [2] reported a relative temperature difference between hands that approaches significance (p=0.052) in a control experiment, where participants watched their own hand being stroked. It is not stated in which direction this trend in temperature difference goes or how large it is, so it is impossible to assess whether this trend also indicates a dissociation of the two measures. The current results also show no correlation between hand cooling and subjectively felt ownership. It is in principle possible that also in Moseley et al.’s [2] study, the reported correlation was driven by a third, external factor that caused both the temperature differences and the differences in subjective vividness, which can happen if results are pooled across conditions. This would then speak further against a direct link between subjectively felt ownership and temperature changes. Unfortunately, none of the other cooling studies report correlation analyses that could help answer this open question. 

We conclude that the cooling effect in the RHI is, at the very least, modulated or catalyzed by still unknown external factors. One should therefore be careful not to rely too heavily on this physiological correlate as an indicator of changes in subjectively felt body ownership (cf. [13], for a similar discussion of proprioceptive drift). Only further research with well-controlled stimulation procedures will clear up how the different measures and modulating factors in the RHI interact and what the generative mechanisms and function of the fascinating cooling effect are.

## Supporting Information

File S1 **Results from Experiments 1, 2, and 3 in a zipped File S1.** The file Table S1 contains the ANOVA tables from statistical tests. Data S1 contains the results in Matlab binary format (.mat). The file Text S1 explains the interpretation of the different variables that are part of the .mat file Data S1.
(ZIP)
