# Correction: The Human Touch: Skin Temperature during the Rubber Hand Illusion in Manual and Automated Stroking Procedures

**DOI:** 10.1371/journal.pone.0189567

**Published:** 2017-12-07

**Authors:** The PLOS ONE Staff

The attribution for Fig 1A was incorrectly omitted. The publisher apologizes for this error.

Please see the complete, correct Fig 1 caption here.

**Fig 1:**
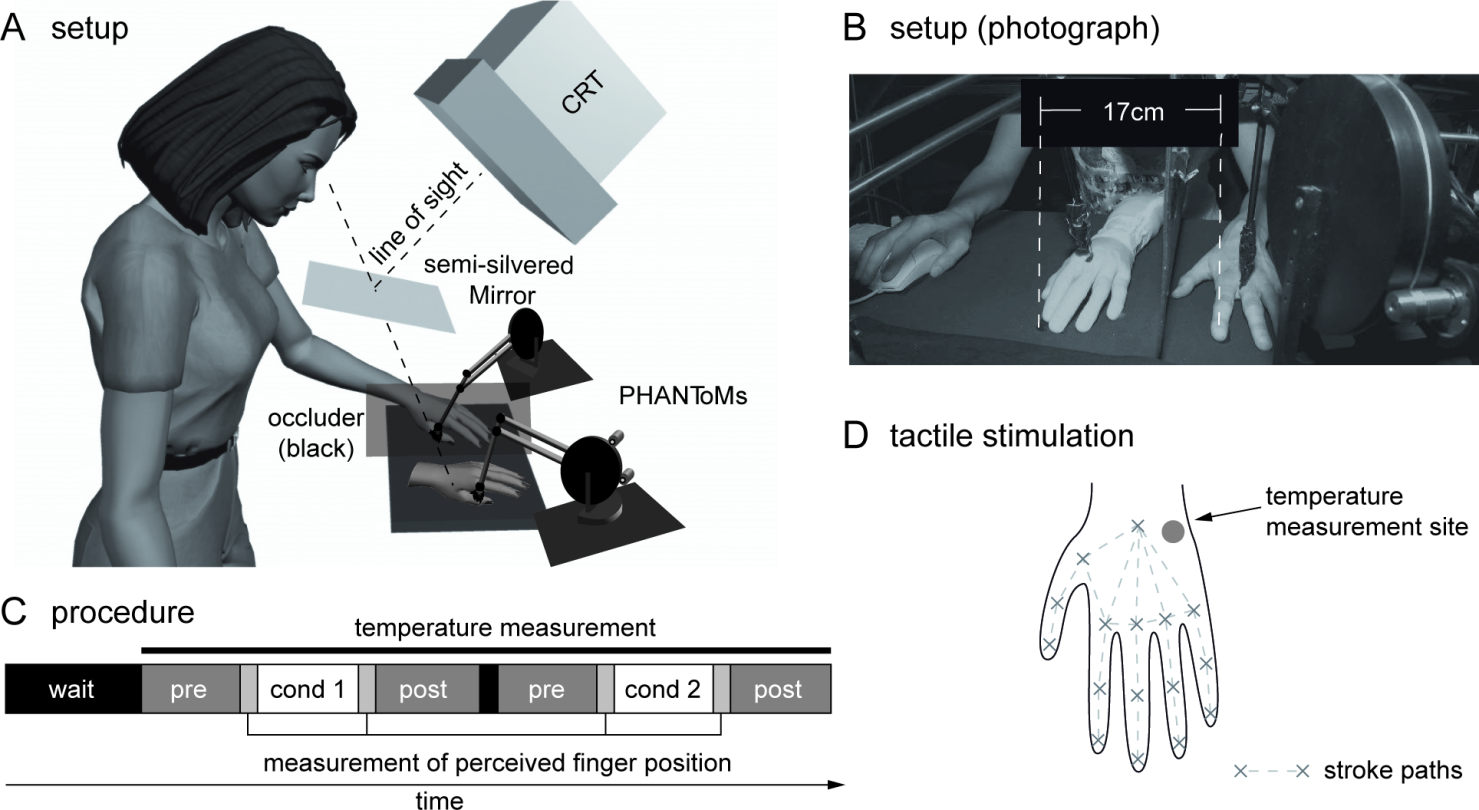
The RHI setup used in the study. Diagram (A) and photograph (B) of the setup. Two PHANToM^TM^ force-feedback devices with paintbrush endings stroke the participant's occluded hand and the visible rubber hand. For testing proprioceptive drift, a probe dot is projected into the visual field using the CRT monitor and a semi-silvered mirror. The participant in (B) has given written informed consent, as outlined in the PLOS consent form, to publication of their photograph. C: The experimental procedure. D: Calibration points and lines along which strokes were applied, as well as temperature measurement location. Image for Fig 1A is courtesy of Rohde M, Di Luca M, Ernst MO (2011) The Rubber Hand Illusion: Feeling of Ownership and Proprioceptive Drift Do Not Go Hand in Hand. PLoS ONE 6(6): e21659. doi:10.1371/journal.pone.0021659.
